# Supplementary material for: Label-free automated three-dimensional imaging of whole organs by microtomy-assisted photoacoustic microscopy
Source: Nat Commun. 2017 Nov 9;8:1386. doi: 10.1038/s41467-017-01649-3 (PMC5680318; doi:10.1038/s41467-017-01649-3)
Supplement: Supplementary file 1 — Supplementary Information [file 41467_2017_1649_MOESM1_ESM.pdf]

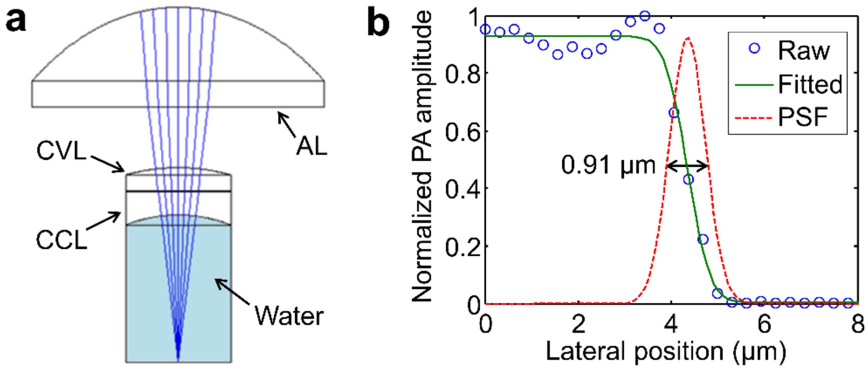

Supplementary Figure 1. Resolution of mPAM. (**a**) Zemax design of the optical objective for focusing UV light into water. AL, aspheric lens; CCL, concave lens; CVL, convex lens. (**b**) By imaging a sharp edge and fitting the data to the error function, the system's point spread function (PSF), which is a product of two orthogonal line-spread functions, is obtained. The lateral resolution of mPAM, defined by the full-width at half maximum of the PSF, is 0.91 µm.

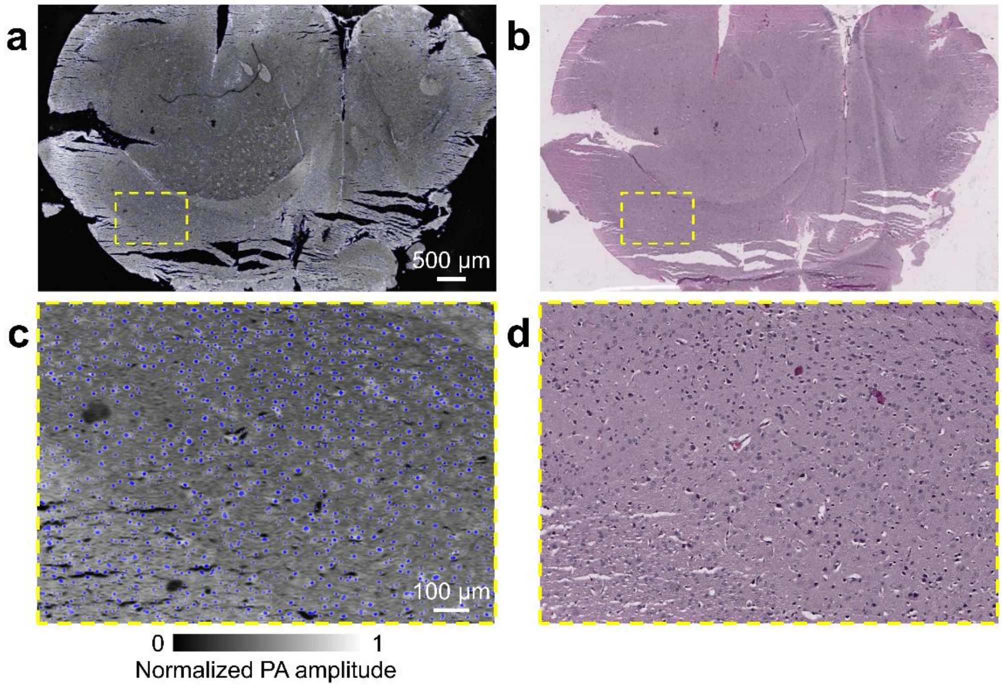

Supplementary Figure 2. Imaging of a paraffin section of a mouse brain. (**a**) Label-free mPAM image, where the cell nuclei are enhanced by a Hessian filter and marked in blue. (**b**) Optical microscopy image acquired after H&E staining. (**c**),(**d**) Close-up images of (**a**) and (**b**), respectively, corresponding to the yellow dashed regions in (**a**) and (**b**). The nuclei are clearly resolved by mPAM.

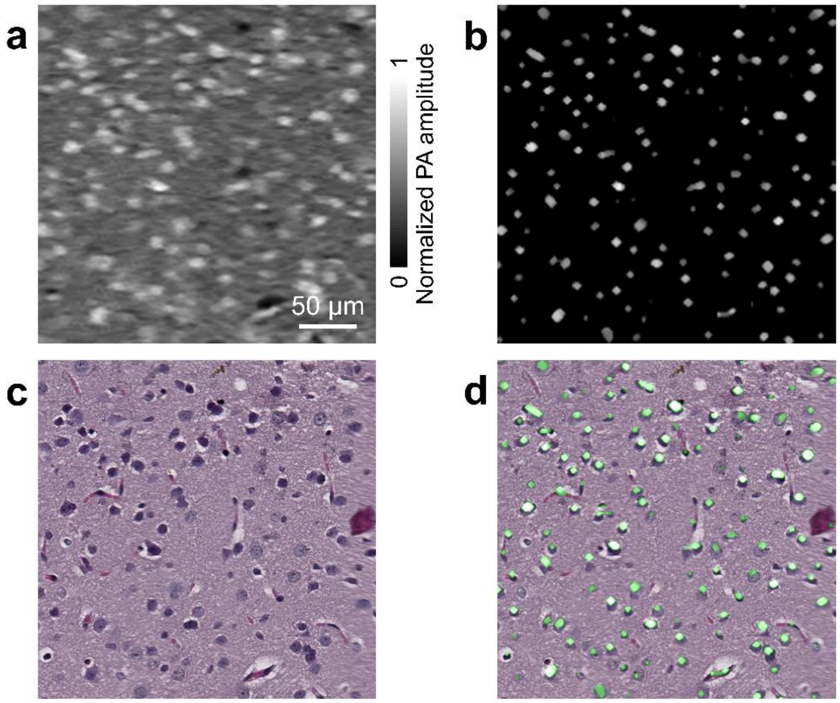

Supplementary Figure 3. Comparison between mPAM and H&E images of a paraffin mouse brain section. (**a**) Label-free mPAM image. (**b**) Nuclei extracted from (**a**) by a Hessian filter. (**c**) Optical microscopy image acquired after H&E staining. (**d**) Superimposed image of (**b**) and (**c**), with (**b**) pseudo-colored in green.

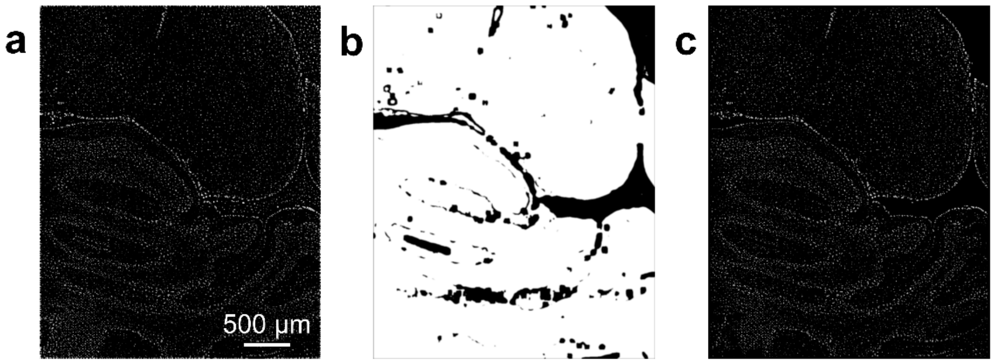

Supplementary Figure 4. Extracting nuclei from label-free mPAM images of a mouse brain embedded in a paraffin block. (**a**) **Fig. 2c** processed by a Hessian filter. (**b**) Nuclear mask calculated from **Fig. 2c**, which separates tissue (bright) from paraffin (dark). (**c**) Nuclear image obtained by masking (**a**) with (**b**).

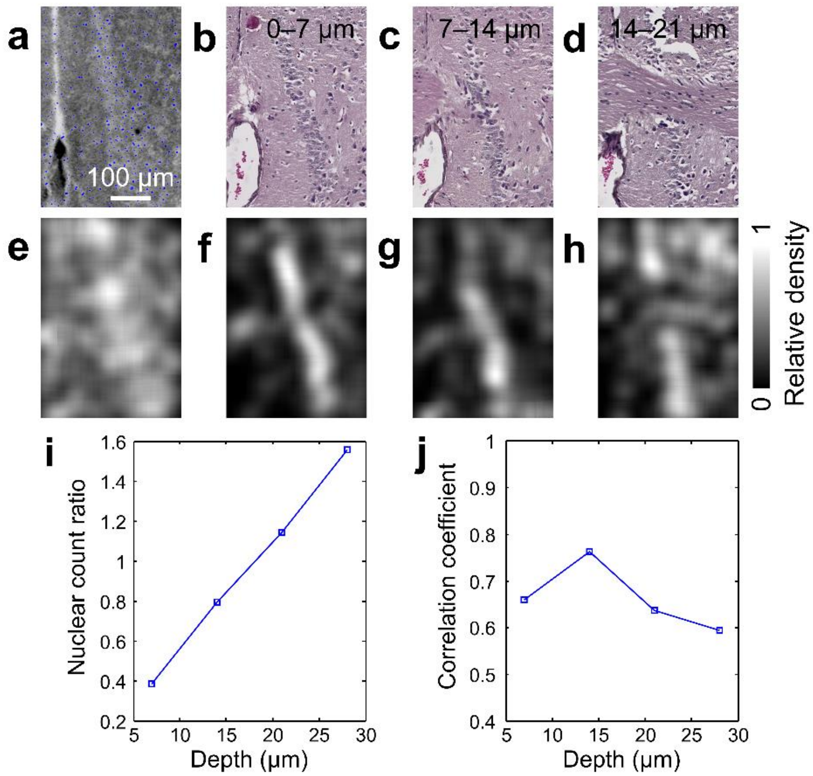

Supplementary Figure 5. Imaging depth of mPAM in a tissue block. (**a**) mPAM image of a paraffin block surface, with nuclei marked in blue. (**b**)–(**d**) H&E images of paraffin sections sliced from the block surface in sequence, each with a 7 µm thickness. (**e**)–(**h**) Nuclear density maps of (**a**)–(**d**), respectively. (**i**) The ratio of the nuclear count in the H&E images within the given depth range to that in the mPAM image. (**j**) The correlation coefficient between the nuclear density map of the H&E images within the given depth range and that of the mPAM image.

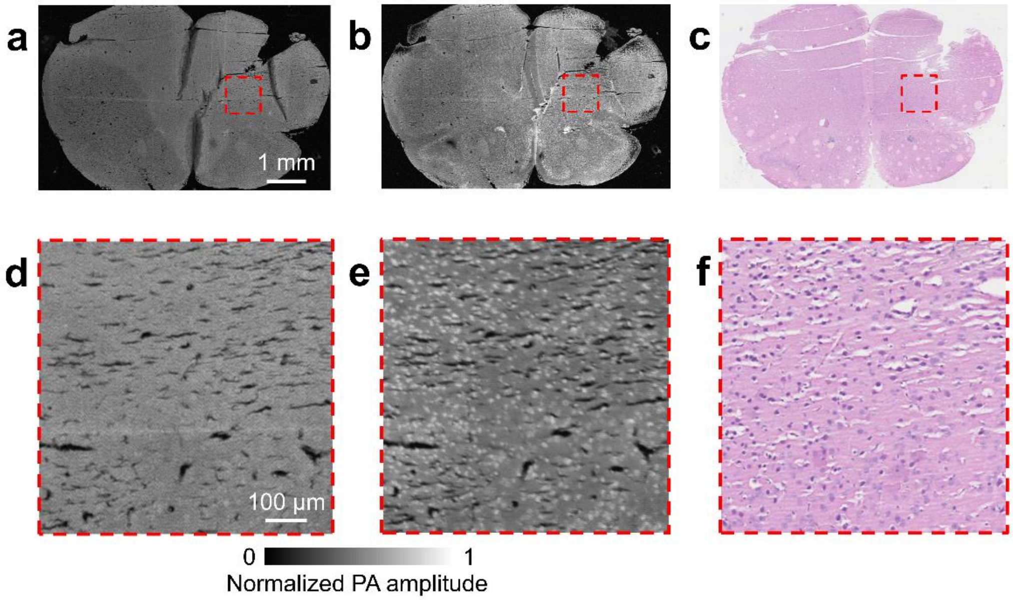

Supplementary Figure 6. Imaging of paraffin-embedded and deparaffinized sections of a mouse brain. (**a**) Label-free mPAM image of the paraffin section. (**b**) Label-free mPAM image of the deparaffinized section. (**c**) Optical microscopy image of the adjacent section after H&E staining. (**d**)–(**f**) Close-up images of (**a**)–(**c**), respectively, corresponding to the red dashed regions in (**a**)–(**c**). The mPAM image of the deparaffinized section shows cell nuclei clearly without resorting to image processing by Hessian filter.

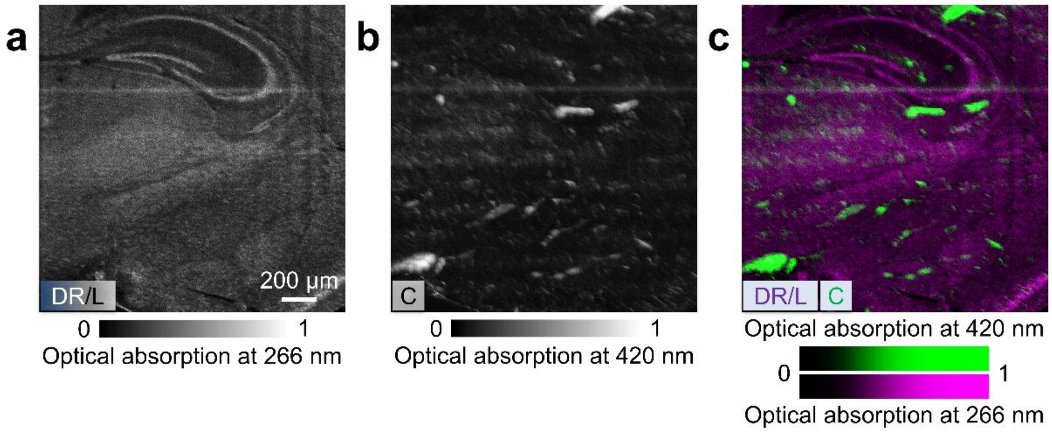

Supplementary Figure 7. Imaging of an agarose-embedded mouse brain section by mPAM with dual wavelengths. (**a**) Label-free mPAM image with 266 nm laser illumination, which mostly shows DNA/RNA (DR) and lipid (L) contrasts. (**b**) Label-free mPAM image with 420 nm laser illumination, which shows cytochrome (C) contrast. (**c**) Overlay image of (**a**) and (**b**), where pseudo colors are used to illustrate the optical absorption color contrast of the biomolecules.

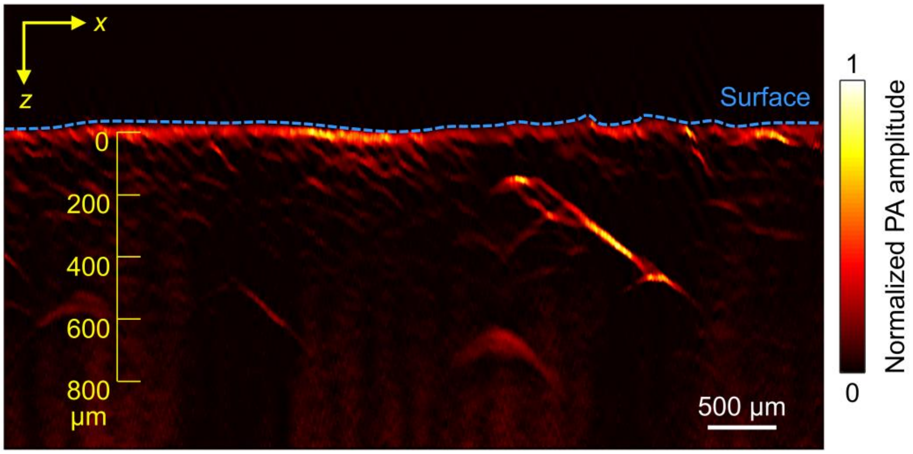

Supplementary Figure 8. Representative *xz* projected 1 mm thick mouse brain image acquired over 6.0 x 0.1 mm^2. The blue dashed line outlines the surface of the mouse brain. With 420 nm light illumination, the deepest cytochrome contrast based structures are ~800 µm in depth measured from the mouse brain surface.
