# Supplementary material for: Label-free automated three-dimensional imaging of whole organs by microtomy-assisted photoacoustic microscopy
Source: Nat Commun. 2017 Nov 9;8:1386. doi: 10.1038/s41467-017-01649-3 (PMC5680318; doi:10.1038/s41467-017-01649-3)
Supplement: Supplementary file 3 — Description of Additional Supplementary Files [file 41467_2017_1649_MOESM3_ESM.pdf]

Description of Supplementary Files

File name: Supplementary Movie 1
Description: An mPAM volumetric image of a paraffin-embedded mouse brain with an imaging volume of 3.8 mm by 3.0 mm by 2.0 mm. The volumetric image was obtained by directly stacking 101 coronal sections with a 20 µm section thickness. The video shows coronal, transverse, and sagittal views, followed by stepping through the volumetric image from different positions and view angles. The cell nuclei are extracted and shown as yellow bright dots.

File name: Supplementary Movie 2
Description: A series of 56 coronal sections of an agarose-embedded mouse brain imaged by mPAM. The imaging field-of-view is 9.5 mm by 7.5 mm. A 3D mouse brain model is shown on the left (reprinted from Elsevier, Vol. 53, Johnson, G. A., Badea, A., Brandenburg, J., Cofer, G., Fubara, B., Liu, S. & Nissanov, J., Waxholm space: an image-based reference for coordinating mouse brain research, 365–372, copyright (2011), with permission from Elsevier)[33], with a red line indicating the relative position of the current coronal section in the entire mouse brain.

File name: Supplementary Movie 3
Description: Close-up images of a row of a representative agarose-embedded mouse brain sections imaged by mPAM in coronal view. The 5X close-up scanning window has a size of ~1.25 x 1.25 mm^2.

File name: Supplementary Movie 4
Description: An mPAM volumetric image of a paraffin-embedded mouse lung with an imaging volume of 1.5 mm by 1.0 mm by 1.2 mm. The volumetric image was obtained by directly stacking 61 coronal sections with a 20 µm sectioning thickness. The video shows the coronal view. The cell nuclei are extracted and shown as yellow bright dots.
